# Retrospect of the Medical Sciences

**Published:** 1843-10-28

**Authors:** 


### ADULTERATION OE COMMON SALT AT PARIS.

Three years ago, it was found that of three thousand samples of common salt taken from the druggists of Paris, more than a tenth part was adulterated. The same fraud has just been discovered among a number of grocers. On making the visits enjoined by the law (which ought to take place in the other towns of France likewise,) it was found that samples showed traces of copper, and were adulterated with a large proportion of impure carbonate of soda (*sel de varech,*) containing iodine. Three specimens contained small crystals of a salt of copper.—*Lon. Med. Gaz. from Gazette Medicale*, August 26, 1843.

### A NEW KNIFE FOR THE OPERATION OF CATARACT.

BY JOHN SCOTT,
Surgeon to the London Hospital, &c.

"The back of the knife describes a sixth part of the circumference of a circle, the radius of which is ten lines. The chord of the arc formed by the back of the knife is, of course, also ten lines in length, being equal to the radius of that circle. The knife is two lines in width at the heel, whence it gradually tapers to the point; it also increases uniformly in thickness, as well as in width, from point to heel." There are four plates to the treatise, the first of which gives a bird's-eye view of the whole matter. A circle is described to represent the globe of the eye, and the new knife is applied to the part corresponding to the cornea. In addition to the points we have already noticed as occuring in the text, the important particulars of the knife having its convex edge sharp on cutting, and its concave edge the reverse, are marked in the diagram. Now for its application. Mr. Scott most justly observes, that the great objection to the knives of Wurzel and Beer are, that they act both as a wedge and a cutting instrument at the same time, in consequence of which the force applied in thrusting them through the anterior chamber tends to turn the eye inwards "to the nasal canthus of the orbit, whereby the inner side of the cornea is obscured from the view of the operator; he is unable to puncture it close to its sclerotic margin, and in consequence, the section is too small for the escape of the cataract." To correct this tendency to inversion, when the wedge knives are used, pressure is often had recourse to on the nasal aspect of the globe, and between the two stools, it often has happened that the lens, vitreous humor, and all, have come to the ground—a feat which has been performed before now in simpler operations than those for cataract. "Scott's knife" is intended to make the necessary section of the cornea for the exit of an opaque lens, without being open to the faults of the other knives which we have mentioned, and at the same time with greater security to the iris than they afford. Those proposed objects in the construction of the instrument are thus numerically stated by our author:—

"1. That it shall be of sufficient length to traverse completely the anterior chamber, and divide the nasal margin of the cornea.

2. That it shall increase in width and in thickness from point to heel, enough only to prevent the escape of the aqueous humor in its transit across the anterior chamber, but that its width shall have no reference to the dimensions of the section that is to be made, as that circumstance, I conceive, has occasioned all the difficulty of its introduction, and the chief danger of the operation.

3. That it shall be of such a shape and figure that when introduced in the middle of the temporal margin of the cornea, and carried across the anterior chamber, it shall readily puncture the nasal side of the membrane; and when placed in this situation, the cutting edge shall be so far beyond the pupillary margin of the iris, and opposed to so large a portion of its anterior surface, as will prevent its escape beneath the edge of the knife to endanger its division in making the section of the cornea.

4. That when the section of the cornea is thus about to be made, the edge of the knife shall be opposed only to the margin of the section on either side, and not to an extensive portion of its internal surface, whereby its division would be attended with difficulty, as is the case in using Beer's knife."—*Provincial Medical Journal.*

### NARROW ESCAPE OF A DRUGGIST.

A Druggist was lately summoned before the police tribunal of the Seine, for having infringed the 34th article of the law of the 21st Germinal of the year XI, which enacts that poisons are to be kept in a separate place, and to be locked up, under a penalty of 3000 francs. The court was of opinion, however, that the sale of the poison was necessary to bring the offence within the meaning of the enactment; and as this had not occurred in the case before them, they merely put into execution the 471st article of the penal code, and adjudged the druggist to pay a fine of five frances, and the expenses.—*Lon. Med. Gaz. from Gazette Medicale*, Sept. 22, 1843.

### RETROSPECT OF THE MEDICAL SCIENCES.

### ON THE [ALLEGED] CURE OF PULMONARY CONSUMPTION WITH NAPHTHA.

BY JOHN HASTINGS, M. D.

Dr. Hastings states that the reason which induced him to deviate from that line of medical practice, which has so universally, and for so long a time, been in vogue, for that which he now brings forward, was the fatal termination of all cases, whatever was the treatment adopted, during an experience of upwards of twenty years.

From the greasy nature of tubercle in its crude state, Dr. Hastings concluded that carbon entered largely into its formation, and that its composition had a striking resemblance to fatty matter. Among the changes in the earlier stages of pulmonary consumption, the disappearence of the fat is about the most remarkable ; in consequence of this loss of fat, the author determined to employ those compound agents, rich in carbon and hydrogen, which had not been previously used in medicine; "not with the idea that they would make up the deficiency which the system had sustained in the progress of the disease, but that such a change would by that means be introduced into the constitution as would act upon the forces of the organism at the point of departure from health, whether that took place in the stomach, blood, or elsewhere;—that change tending to such an affinity in the elements within the body, that the carbon, hydrogen, oxygen and nitrogen, instead of assisting in the formation of products which threaten life, would tend to develope those materials only which are required for the perpetuation of health, and the prolongation of existence."

Accordingly Dr. Hastings was led to employ naphtha as a remedy in pulmonary consumption. Many different compounds pass under this name, but the kind of naphtha termed *pyro-acetic spirit*, obtained by the destructive distillation of an acetate, generally of lead or lime, and in its outward form scarcely distinguishable from pyroxilic spirit, is the species which is considered to be the best suited for this purpose.

The following is the way in which Dr. Hastings employs the remedy.

"I administer naphtha three times a day, in doses of fifteen drops for an adult, mixed with a table spoonful of water, which is proportionably decreased according as the patient approaches youth. After the second or third day, I increase the dose by about one-fourth ; regulating its increase or decrease, according to the increase or decrease of nausea, sickness, or any other untoward symptom arising out of its use. As the disease advances, I increase the dose to forty and even fifty drops, and administer it four times a day, instead of three times.

"The succesful use of naphtha as an internal remedy, induced me to try its effects by inhalation, to which I was the more inclined from the results of the following experiments:—

"1st.—A little naphtha having been put into a bent tube, resembling the capital U, some expectorated matter was poured upon it, which had been determined with the microscope to be rich in globules of tubercle. Gentle heat was then applied, and the naphtha driven off, when the super-imposed secretion presented a mere shapeless mass of animal matter, the globules having entirely disappeared.

"2d.—Some tuberculous secretion, highly charged with globules of tubercle, was placed under the field of the microscope, and a drop of naphtha added, when an immediate disappearance of the globules ensued, leaving behind a mass of the same character as in the former case. The frequent repetition of this experiment invariably led to the same result.

"3d.—Some tuberculous secretion of the lungs was put into a portion of the intestine of a child, and placed over a wide-mouthed bottle, which contained a small quantity of naphtha, between which and the intestine a clear space of three inches remained. A spirit lamp was then placed under the bottle, and a very gentle heat applied until slight ebullition took place, which was continued for an hour. The contents, when removed from the intestine and examined with the microscope, presented the same appearance as described in the previous experiments.

"Considerable benefit resulted from the inhalation of naphtha, in lessening the difficulty of breathing, in the most advanced cases, in rendering muscular efforts less painful and fatiguing, and in general alleviation of those symptoms which distress the consumptive patient. The expectoration is not unfrequently rather increased immediately after the inhalation of naphtha, but the cough has changed for one of a milder character. Improvement was generally observed to follow that kind of inhalation which was performed with little exertion. It may be employed several times in the day, unless it produces nausea and sickness, when its use should be suspended ; and on its being resumed, in such cases, it should be applied for a shorter period. When there is spitting of blood its use is not admissible."

"Almost immediately after naphtha has been administered, an occasional rising of the medicine is perceptible in the mouth and throat, similar to that which occurs after a dose of castor oil. This is sometimes followed by nausea, and now and then vomiting supervenes. At other times it acts, but much more rarely, as an aperient. But when these effects occur, they usually subside in a day or two. It not unfrequently produces a glow in the region of the stomach, which extends over the chest and creates a sensation of cheerfulness and a greater freedom of breathing. I appears deserving a high rank among tonics: for in most of the cases in which it has been employed, a natural appetite was in a short time established. No remedial agent that I am acquainted with possesses such power over the colliquative perspirations of pulmonary consumption; as a few doses, in most instances, appeared sufficient to effect their removal. Another fact worthy of remark, is the absence of diarrhœa in all cases, which may be accounted for upon the supposition that tubercular deposite ceases to take place in the mucous track of the intestines. And even in those cases where diarrhœa in the first instance, existed, it readily yielded to naphtha treatment. The thirty-third case is a good example of this remark. Headache, particularly when the bowels are confined, is sometimes the effect of the naphtha treatment, and if aperients fail to give relief, a mustard poultice should be applied to the back of the neck, or a few leeches to the temples, or behind the ears. It will, however, be very seldom necessary to suspend the employment of the naphtha from this cause."

According to Dr. Hastings, from the very first moment he employed naphtha in pulmonary consumption up to the present time, it has been so successful in his hands, that he has no doubt it will be found, if used judiciously, to be little less than a specific in the earlier stages of the disease. And, from what he has more recently observed, he is most sanguine that even in the latter stages of the disease, a restoration of health may be generally calculated upon.

"The progress of improvement in the physical signs, when sufficiently marked, invariably commenced with an amendment of the sounds arising from percussion. In no case did they appear to begin with auscultation; consequently I am induced to form the opinion that, as diagnostic signs, those derived from auscultation take precedence of those from percussion, or, in other words, that changes such as prolonged expiration, and very slight, feeble and harsh respiration, may be detected by auscultation, when the sound elicited by percussion is not sufficiently appreciable to lead to any useful conclusion considered apart from auscultation."

Many cases are narrated in which the naphtha was employed.

If experience proves these statements, Dr. Hastings will, no doubt, be considered a benefactor to the human race.—*Medico-Chirurgical Review*, Oct. 1843.

*Varus Mentagra and Gutta Rosea, the Sycosis Menti and Acne of Willan, Treated with sulphate of iron externally.* By W. Dauverger.

Of all the modes of treatment recommended for these obstinate affections, the following appears to have been the most useful in the author's hands.

The sulphate of iron in different forms is the most efficacious local remedy for the pustular inflammation of gutta rosea, and mentagra.

It is used in solution, either by bathing the part affected, or by applying linen dipped in it, or by sprinkling the ulcerated parts of the mentagra with a mixture of charcoal and sulphate of iron. This mixture need not be finely levigated, for it then forms a crust too easily removed by washing the beard. In spite of his previous opinions, M. Dauverger also tried a pommade of sulphate of iron, but was obliged to give it up. The following are the formulæ employed by him.

No. 1.—Sulphate of iron twenty-five grammes. Distilled water two hundred grammes. Dissolve.

No. 2.—Sulphate of iron fifty grammes. Distilled water two hundred grammes. Dissolve.

No. 3.—*Ferro-carbonic powder.* Sulphate of iron ten grammes. Charcoal thirty-five grammes. Powder and mix.

The author first treats the inflammatory symptoms with emollients; when he thinks them sufficiently reduced, he orders the patient to bathe the part twice a day with two glasses of warm water containing one or two spoonfuls of the solution No. 1. A quarter of an hour afterwards, he prescribes a local bath of an emollient decoction; and afterwards, if possible, the application of a poultice of the same kind. When no further improvement takes place under this treatment, he has recourse to No. 2, which is twice the strength of the former, and proceeds in the same way. The author employs general means of treatment at the same time,—*Lon. Med. Gaz. from Gazette Médicale*, Sept. 9, 1843.

CONVENIENT MODE OF ADMINISTERING OIL OF TURPENTINE. BY M. BOUCHARDAT.

The oil of turpentine is very often used internally, and might be employed still more frequently, were it not that the usual formulæ for its administration, such as the terebinthinous either, or Durand's remedy, turpentine emulsion, and the different terebinthinous mixtures, all fail in masking the disagreeable taste of this medicine. The following, however, is the form of an electuary which M. Bouchardat assures us may be administered with the greatest ease.

Take of gum acacia, ten grammes; mix it with ten grammes of water; add of white honey, fifty grammes; oil of turpentine, fifty grammes; carbonate of magnesia, q. s. Make a soft electuary.

The dose is from 2 to 10 grammes (36 to 180 grains) a day, in unleavened bread.

In some cases a little opium, or from 10 to 20 drops of Rousseau's laudanum, may be added to the mucilage in the above mixture.—*Lon. Med. Gaz. from Bulletin Général de Thérapeutiqué*, Sept. 22, 1843.

ON THE FREQUENCY OF CRIMINAL ABORTION.

We have observed that the newspapers have been unusually full of late of cases in which the attempt to produce criminal abortion has come under the cognizance of the legal authorities, in consequence of the injurious or fatal consequences to the unhappy females involved; but we cannot help thinking, both from our own experience and from that of many of our professional friends, that the number of instances in which this crime is perpetrated successfully, and without detection, is out of all proportion to the comparatively small number of cases that are found out and punished.

But this, like many other evils, is much more easily lamented than remedied. So long as the existing state of civilization places restrictions on early marriage—so long as the indiscretion of youth, and the habitual sensuality of maturer years, have their force—and so long as the loss of her fair fame and social position hang over the head of the guilty female—in other words, so long as human nature remains what it is, the attempt will continue to be made to conceal and destroy the fruit of sexual transgressions. And our own experience leads us to believe that the attempt is by no means so difficult as some writers seem to respresent it. We find Dr. Guy, for instance, in his excellent "Principles of Forensic Medicine," stating as the result of his investigations, "that there is no one medicine which can be depended on as a means of producing abortion; that, if given in doses short of those which would destroy the life of the mother, they would almost certainly fail of accomplishing their purpose; that, when they do succeed, they place the life of the mother in jeopardy, and often sacrifice it; and that, for every case in which the mother escapes, there is, probably, one at least in which the mother and her offspring both fall a sacrifice, and one in which the mother dies, the child remaining uninjured in her womb.

"The fact is, that none but poisons, or medicines administered in poisonous doses, can be expected to produce abortion in any case, unless the predisposition to abortion is already very strong. When such predisposition does not exist, the mother is much more likely to fall a sacrifice, whilst the child remains intact in the womb, or is even born alive, than the child to be expelled, and the mother to survive; in other cases both the child and offspring will perish."

Now, we cannot help thinking that the danger and difficulty are both considerably overstated by Dr. Guy, and other writers on the subject, provided that the means are used cautiously, and by a practised hand. Instances are common enough, within the circle of our own professional observation, in which young women have confessed that they have been pregnant, but that they have taken something which has made them *right* again, as they express it; and we have heard of a French hag, living somewhere near Marylebone-lane, who enjoys no small share of fame for her success, the means which she employs consisting in the daily administration of the oils of pennyroyal and savine, with a violent cathartic at intervals of two or three days.

We may be asked, what is the use of calling attention to these circumstances, degrading as they are, but almost irremediable? We answer, that there may be one small class of cases in which the crime is committed in ignorance of its real nature and enormity. More than one accoucheur has a story to tell of an innocent-looking girl requesting to be disburdened of the fruits of clandestine sexual commerce, with as much coolness and *naïveté* as if she wished to be relieved from a troublesome tooth; and when remonstrances are made upon the wickedness of such a proceeding, the common reply is, that "there can be no harm in doing so before the little thing is alive, but that, at a later time, it would be very wicked, and not to be thought of." In fact, the opinion that the fœtus, for the first two or three months, is an insensate vegetable mass, but that it becomes *quick* at a definite period, was once held true in medicine, and adopted as the principle of legislation, and, although now discarded, it still lingers, like a thread-bare garment, amongst the lower orders of society; and it serves, perhaps, to quiet the scruples of some few of the less hardened offenders. Here, therefore, is an opening, although we fear a very limited one, for the diffusion of medical knowledge, as a means of diminishing mortality and preventing crime.—*Prov. Med. Jour.*

### DRS. HAPLIN AND JOHNS ON PUERPERAL CONVULSIONS.

In the September number of the "Dublin Journal," are two papers by the above named gentlemen on this prevalent and alarming disease. Dr. Johns wishes to call particular attention to the fact, that however sudden the seizure may apparently be, certain premonitory symptoms are seldom or never wanting; and that if these are met with prompt and judicious treatment, the attack may generally be prevented. We may remark, that Osiander states that a tumid state of the face and superior extremities almost always precedes the attack, and it is this symptom which Dr. Johns thinks so important as a precursory sign. "It is familiarly known," he observes, "that during the last months of pregnancy, swelling and œdema of the inferior extremities very frequently occur, and these symptoms are justly considered as a harmless complication; but if a similar affection is observed to attack the superior parts of the body, as the hands and arms, the neck and the face, the case will then require a more close and accurate examination: for if in combination with this symptom their exist headache, weight, or giddiness in the head, ringing in the ears, temporary loss of vision, severe pain in the stomach, with flushed face, there will be risk of convulsions; a risk that will be converted into certainty, if—1st, The woman is pregnant for the first time, or has similarly suffered in former pregnancies; 2nd, When the head of the child presents as in ordinary labours; and, 3rd, Where the woman is of a full and plethoric habit." Dr. Johns subsequently gives twenty-one cases of convulsions, actual or threatened, occuring within the last two years, in eighteen of which œdema of the face and superior extremities was present. Dr. Haplin comes to the following general conclusions:—

1. Puerperal convulsions occur most frequently in first pregnancies; the ratio being six in seven.
2. Amongst the predisposing causes, the age of the patient seems to have much influence. In one-fourth of the recorded cases, the women were above twenty-eight years old.
3. There is no decided premonitory symptom invariably present.
4. The presentation is almost invariably natural.
5. They are always attended with danger both to the mother and child. One-fourth of the mothers, and two-thirds of the children perish.
6. In the treatment of this disease, copious blood-letting, early resorted to, and rapidly effected, is a measure of the first importance.
7. Active purgatives should be administered, either by the mouth or in injections, until the stomach and bowels are thoroughly cleansed of offensive matter.
8. Should those measures fail in controlling the disease, the uterus should be emptied of its contents as early as the state of the parts will admit of its being done without violence.
9. The natural effects are frequently sufficient to effect the delivery.
10. When interference is required, the forceps or vectis, are to be preferred in many cases to turning or the perforator; as
11. With the perforator the children are necessarily destroyed; and
12. Turning the child is attented with great danger to the mother, as five out of the seven of those on whom it has been practised have died.—(*Collins.*)
13. The pathological appearances are frequently insufficient to account for the violence of the symptoms that characterise this disease.—*Prov. Med. Jour.*

### DELIRIUM TREMENS.

BY HENRY JOHNSON, M. D.

As a substitute for delirium tremens, Dr. Johnson proposes the term Hypophrenitis, "as more expressive of its peculiar nature than the old one. Independent of my own observation, it would be easy to adduce authorities to prove that delirium tremens is, in many cases, neither more nor less than phrenitis. The phenomena during life, and the appearances after death, show this. It does not, indeed, bear the active and decided treatment which ordinary phrenitis endures and requires. This is because it arises from a peculiar debilitating cause, and in a constitution weakened by excess. The inflammatory symptoms which may have appeared at first, soon give way to those of *depression* and *irritation*. Hence the utility of sedative and narcotics, or a combination of these with stimulants. I have, therefore, named this disease *hypophrenitis*, as a less acute form of the disorder, with greater tendency to debility, and it is, I think, correctly placed between phrenitis and the next disease (insanity.) Its affinity to the former is made evident, by the frequent occurrence of cases which require the treatment of phrenitis; and its relationship to the latter is shown by its occasional degeneration into insanity. In some instances the inflammatory symptoms are slight, and those of debility, and what has been called cerebral irritation, are more prominent. It is chiefly distinguished from phrenitis by the presence of tremor, by the soft, quick pulse, the absence of intolerance of light, the profuse perspirations, the nature of its cause, the peculiarity of the delirium. The countenance is anxious and depressed; the mind is continually and painfully engaged with the customary pursuits and occupations of the patient.

The majority of cases of this disease recover in a few weeks. When fatal, the signs of morbid action within the head are not very marked, but enough to show the character of the disease. The arachnoid is found slightly opaque, the pia mater injected, and effusion of serum within the ventricles."

The obscurities which have so long beset the subject of delirium tremens, and the diversities of opinion entertained as to its nature and treatment, are, we think, inseparable from the hitherto prevailing systems of medical study, in which the disease was taken as the substance, the patient as the accident; in which inflammation was considered an intruding something, which must be reduced to submission by bleeding and starvation, and in which no room was found for the idea of vascular turgescence and structural change, as consequences of slow misuse of function or disturbance of nutrition; or of their thus being prevented at first by means which would aggravate them if employed subsequently. Hence the confusion of opinions; the conflicting treatment proposed for the same name of a disease; the idea inconceivable that quiet anodynes and nourishment could prevent a state which required subsequently the abstraction of blood, and which left turgid blood-vessels and opaque membranes, as supposed evidence of its inflammatory character. An improving method of study, the restriction of morbid anatomy to its proper purposes, and a more cautious application of abstract terms, will, we trust, before many years have elapsed, clear away some of the apparent opprobrium incurred by the proposal of opposite treatments for the same disease."—*Ib.*

### MORTALITY AFTER AMPUTATION.

The striking fallacy of deductions to the mortality of operations, founded on a short period of observation, is well shown by comparison of the last two tables with those given in the preceding report; for it appears that of 16 amputations performed during the last nine months, 11 were cured and 5 died; whereas, out of 72 cases the number which occurred during four years, 37 were cured and 35 died.

*Statistical Tables of the Royal Infirm. of Edinburg.*

### OBSERVATIONS ON THE MUSCLE OF THE LACRYMAL SAC, OR TENSOR TARSI.

My attention to this muscle, as a part distinct from the orbicularis palpebrarum, was, with most anatomists I presume, first directed by Dr. Horner, of Philadelphia, about the year 1821. On re-examining the muscle at that time, it appeared to me—1st, that many would be disposed to view the tensor tarsi merely as a more or less detached portion of the sphincter. 2dly, What was most evident to myself, its anatomy had not been given correctly by Dr. Horner. From that time to the present I have often had occasion to repeat these remarks, but did not deem the matter worthy further notice. Lately, however, I observe that a very distinguished oculist and surgeon, Dr. W. Mackenzie, of Glasgow, has reviewed the matter in an historical point of view, contesting the discovery of the muscle with Dr. Horner, and restoring it to Du Verney, to whom the merit of having first described it evidently belongs. This called my attention again to the muscle; and as both the descriptions of Du Verney and of Dr. Horner are inaccurate, conveying to the student of anatomy and physiology a false notion of the shape, extent, uses, and connection of the muscle with the orbicularis, I have thought it worth while to submit the following brief observations.

In 1805, Rosenmüller gave a drawing of the muscle, and a method of dissecting it. There can be no objection made to his method of display, but the drawing is very imperfect. He recommends the eyelids to be cut from the edge of the orbits, and laid down over the nose, and of course towards the opposite side: the membrana nictitans comes in view, which is then to be dissected off; below it will be found a quantity of fat, cellular substance, and nervous filaments, with a firm though delicate fascia of cellular membrane covering the surface of the muscle. On removing this with the scalpel, a muscle of the size stated by Rosenmüller comes into view; it arises as if fleshy, and at once from the back of the os unguis, or where that bone meets the orbitar plate of the ethmoid, and proceeding forward and outward (but forward and inward as looked at during the dissection,) soon divides into two portions, proceeding to the inner surface of the tarsal cartilages. Now instead of stopping at the puncta lachrymalia, as described by Rosenmüller, and as is represented in his drawings, and as is described, I presume, by most anatomists of the day, the bifurcated muscle runs on quite to the opposite extremity of the eyelids, thus doubling that part of the sphincter coming from the upper and lower part of the tendo oculi, and forming in itself the more immediate and most powerful part of the sphincter of the eyelids. This at least is what I have often seen.

By this arrangement the sphere of action of the muscle, when fully developed; is much more extended than is generally supposed. It supports the eyelids, draws them forcibly towards the inner canthus, and in the most efficient way, being bound down by cellular substance to the lacrymal sac, retains the eyelids in apposition to the eyeball, which no part of the orbicularis, as it is now arranged, could have done. Relaxation of the eyelids may depend, I imagine, on a relaxed condition of this muscle; and that peculiar and contracted condition of the eyelids, which every surgeon must have observed in some persons subject to ophthalmia with inversion of the eyelids, in all probability may depend occasionally on a spasmodic state of this muscle.

I ought to have added, that there is a very good description of the tensor tarsi in the French translation of Meckel's Anatomy.—*Lond. Med. Gaz.*

### A CAUTION RESPECTING NITRIC ACID.

We not unfrequently see prescriptions in which five or six minims of nitric acid are ordered for a dose, diffused in an ounce or an ounce and a half of fluid. This is a strong dose when the acid is of sp. gr. 1.4, as was the case with the acid generally used until lately. But since the publication of the remarks of Mr. Phillips on the subject, which drew attention to the fact that the acid ordered in the Pharmacopœia is of sp. gr. 1.504, the manufacturers have supplied the article according to the correct standard; and the circumstance not having been sufficiently made known in the medical profession, patients have sometimes suffered from the inconvenience of taking a dose considerably stronger than was intended. In such cases we conceive it to be the duty of the pharmaceutical chemist to impart that information to the prescriber which shall enable him to regulate the dose accordingly.

The maximum dose of acidum nitricum dilutum is stated in the Pharmacopœia to be forty minims (equal to four minims of the strong acid;) and we have no hesitation in stating that this quantity is quite sufficient, *unless largely diluted*, to act injuriously on the enamel of the teeth. On reference to some other authorities, we see the dose of strong nitric acid stated as "from five to ten minims;" and on this account might have felt a delicacy in animadverting on the subject, if we had not repeatedly heard serious complaints from patients. An instance lately occurred in which six minims were taken in an ounce of fluid, three times a day. In the course of two or three days the teeth were found to be seriously injured, to the great annoyance of the medical attendant, who was not aware that he had ordered more than might be taken with perfect safety. In all cases in which it is desirable to administer large doses of this powerful acid, care should be taken to dilute it sufficiently, and the patient should be directed to rinse the mouth with water, or a solution of carbonate of potash, immediately after having taken each dose. These precautions should never be neglected, by those who consider the preservation of a good set of teeth of any importance.

We may also observe that the strong nitric acid should never be used in dispensing in small quantities, as it is impossible to measure a few minims with so much accuracy as a proportionate quantity of the diluted acid.

Nitric acid of sp. gr. 1.504, always contains a considerable portion of nitrous acid, which gives it a pale yellow colour. The action of light and air occasions the liberation of oxygen, and the consequent conversion of a further portion of nitric into nitrous acid. According to M. Millon this is the case, more or less, with commercial nitric acid of all densities, but more particularly when highly concentrated; consequently, the Acidum Nitricum P. L., is not a convenient preparation for general use, and should be kept in the dark, and not unnecessarily exposed to the action of the air by the frequent removal of the stopper.—*Pharm. Journ.*

### FLUID EXTRACT OF SENNA.

PY PROFESSOR CHRISTISON.

Take fifteen pounds avoirdupois or Tinnevelly senna, and exhaust it with boiling water by displacement: about four times its weight of water is sufficient. Concentrate the infusion in vacuo to ten pounds; dissolve in the product six pounds of treacle previously concentrated over the vapour-bath, till a little of it becomes nearly dry on cooling; add twenty-four fluid ounces of rectified spirit (dens. .835;) and, if necessary, add water to make fifteen (16 oz.) pints—the object being that the preparation shall be of such strength that every fluid ounce shall correspond to one avoirdupois ounce of senna. Mr. Duncan, of Edinburgh, generally makes eighty pounds of senna into this extract in one operation. The numbers given are those by which he worked in the first instance. The dose is two drachms for an adult; it very rarely causes griping. It tastes precisely like treacle, and the absence of disagreeable taste is owing to the fact that pure senna has but a feeble mawkish taste, which treacle easily covers.

*Ibid.*

### OBSERVATIONS AND RESEARCHES UPON A NEW SOLVENT FOR STONE IN THE BLADDER.

BY ALEXANDER URE, A. M.

In pursuing some inquiries relative to the treatment of certain forms of urinary disease, my attention was directed to the properties of carbonate of lithia, a substance of which no therapeutic application has been heretofore made. It nevertheless occurs as a constituent of various mineral waters—namely, in those of the Kreuzbrunnen of Marienbad, of Klausen, of the Josephquelle at Bilin, of the Obersalzbrunnen in Silesia, of Lubien in Galicia, of the Kränchen at Ems, and of the Franzensbrunnen at Eger. The four first named waters have, according to Osann, one of the latest and most complete writers on the subject, been found of service in some unhealthy conditions of the urinary organs.

Carbonate of lithia dissolves in water at the ordinary temperature of 60° Fah., to the amount of one per cent. From its sparing solubility it may be said to form the connecting link between the earths and alkalies. It possesses a faintly alkaline by no means unpleasant taste. No opportunity has yet been afforded me of ascertaining whether it passes through the circulation unchanged, although analogy would lead to the supposition that such was the case. It has a remarkable affinity for uric acid, so much so, that if finely pulverised *lepidolite* (a hard siliceous mineral containing three or four per cent. of lithia) be boiled along with uric acid in water, urate of lithia is formed. A fact pointed out by M. Lipowitz, and which has been lately verified by himself.

According to the chemist above mentioned, one part of carbonate of lithia dissolved in water and boiled along with an excess of uric acid, dissolves four parts of the latter, which are held in solution, after cooling. Urate of lithia is indeed the most soluble salt which that acid forms. It crystallises by evaporation in the shape of small grains, which require sixty parts of water at the temperature of 122° Fah. to dissolve them. It contains 14.4 per cent. of lithia.

In order to determine the solvent powers of carbonate of lithia with reference to the uric acid and its compounds, at the common temperature of the human body, I instituted the following experiments:—

A solution of one grain of carbonate of lithia in an ounce of distilled water was brought to a temperature of 98°, and pure uric acid gradually added in minute portions until it ceased to dissolve. This quantity thus taken up, was 2.3 grains. The resulting solution, which remained unchanged the following morning, being saturated with hydrochloric acid, threw down a precipitate of uric acid, amounting to two grains. Now it will be seen, by referring to my paper on the "solvents for calculous concretions," published in the fifth number of the "Pharmaceutical Journal," Vol. I, that one grain of crystals of soda dissolved in an ounce of water, took up only one grain of uric acid—that one grain of carbonate of potash took up 1.4 grains—one grain of borax 1.2 grains—and four grains of bicarbonate of soda 1.1 grains. Hence it follows that the solvent powers of carbonate of lithia is more than double that of carbonate of soda, nearly double that of carbonate of potash or borax, and about eight times that of bicarbonate of soda, which is the active ingredient of the Vichy water.

A human urinary calculus, now on the table of the society, composed of uric acid with alternate layers of oxalate of lime, having been most accurately poised, after being previously brought to hygrometric repose, by digesting in fresh urine and then carefully dried, was placed in a solution of four grains of carbonate of lithia in an ounce of distilled water, and steadily maintained at a blood-heat by means of a water-bath, during five consecutive hours. On being withdrawn, nicely washed, and again dried as before, it was found to have lost five grains weight, which is at the rate of one grain an hour. The calculus is deeply eroded in different parts, but the delicate laminæ of oxalate of lime remain intact, imparting to the surface the appearance of deep etching. The menstruum acquired a pale yellow tinge, and there fell down from it on cooling a light flocculent deposite of urate of lithia, in which silky crystaline tufts could be discerned by help of the microscope It was still alkaline to litmus. Decomposed by means of hydrochloric acid, it yielded nearly three grains of pure uric acid. In another experiment the remaining half of the same calculus being allowed to stand during four hours in two ounces of the natural Vichy water, from the spring called *Hôpital* (containing three grains and a half of carbonate of soda), was found to have parted with two-tenths of a grain of uric acid; while the former portion of the calculus, placed under precisely similar circumstances, at the same time, in a solution of 1.6 grains of carbonate of lithia to two ounces of distilled water, afforded nine-tenths of a grain of uric acid. Thus is demonstrated the very superior solvent agency of the above feeble lithia solution over the Vichy water.

Half a grain of urate of soda (the ordinary basis of gouty concretions or chalk stones) diffused in an ounce of distilled water at the blood-heat, completely dissolved with the addition of one grain of carbonate of lithia the solution continuing limpid and unaltered; whereas, half a grain of the same urate in a similar quantity of water at a corresponding temperature, rests apparently unchanged, as may be seen in the two phials before you. Urate of soda, as pointed out in my paper on gouty concretions, published in Vol. XXIV of the Medico-Chirurgical Transactions, is about as insoluble as uric acid.

It deserves notice, that when fresh healthy urine is rendered alkaline by carbonate of lithia, no deposition ensues.

A very large proportion of the stones which occur in the urinary bladder of man, are composed in whole or in part of uric acid. Of all the various menstrua hitherto recommended, none appears to promise more favorably than the carbonate of lithia, from the promptitude and energy with which in dilute solution it attacks calculi of this description. If by means of injection we can reduce a stone at the rate of a grain or more an hour, as the above experiment would lead us to anticipate, we shall not merely diminish the positive bulk of the calculus, but farther loosen its cohesion, disintegrate it, so to speak, causing it to crumble down and be washed away in the stream of urine. Cases may present themselves in which it may be expedient to conjoin the use of the lithontriptor; but only occasionally and at long intervals. It is the frequency of repetition which renders that instrument so hazardous.

It may be presumed, moreover, that the plan of throwing in a weak solution of this kind, would generally exercise a beneficial influence in obviating irritation, by removing the sharp angular points and asperities of the broken fragments, where the practice of crushing is adopted.

No apprehension need be entertained from the administration of injections if judiciously directed. Sir Benjamin Brodie found that the bladder bore without inconvenience a stream of fluid composed of two minims and a half of nitric acid for each ounce of distilled water. An Austrian surgeon has recently introduced vinegar into the bladder, with excellent success, in an instance of phosphatic calculus. M. Lisfranc, the eminent French surgeon, has used in like manner tincture of cantharides for the cure of enuresis; and I myself have thrown a dilute solution of nitrate of silver into the bladder, with the best effect, in cases of chronic catarrh of that viscus.

Nothing has hindered me from trying the carbonate of lithia but its extreme scarcity. I would, therefore, suggest the importance of its preparation to the Pharmaceutical chemist. The mineral called *spodumene*, which is found in Killiney, near Dublin, contains, according to Stromeyer's analysis, 5.6 per cent. of lithia.—*Pharmaceutical Journal.*

---

URINE IN TYPHUS. BY DR. SIMON, OF BERLIN.

In the second volume of my "Medical Chemistry" I have spoken of a peculiar character of the urine in typhus, to which my attention was first directed by Dr. Schönlein, and is important as well with respect to the development of the morbid process as for the prognosis. This regards the circumstance of the urine becoming alkaline at certain times, which alkalinity lasts for a certain time, after having had at first a strong and acid reaction for some time, and the simultaneous occurrence of sediments, which, according to their nature, may bear a different signification, and whose appearance together with the reaction of the urine may be of considerable import, if correctly appreciated, with respect to the development of the disease. With respect to this matter, Schönlein remarks, that in the regular course of typhoid disease the urine is found at first to be dark, and to have a very acid reaction, whilst subsequently it becomes neutral and then alkaline; lastly, when the disease is tending to improve, the acid reaction again sets in. I had previously made observations which entirely correspond with these; but I have, on the contrary, seen cases where the urine presented quite different properties,—it either continued permanently acid, or else became alkaline, only, however, for a short time, and soon again it resumed the acid reaction; in some cases an amendment took place, but on two occasions death followed. I afterwards followed up my enquiries regarding the urine in typhus with increased attention, and to the two cases previously observed I can now add six new ones, which further confirm the properties just mentioned. In the one case the urine was slightly alkaline on the seventh day after the patient's admission, and continued so, or with a neutral reaction, for seven or eight days, then it again became slightly acid and clear—the patient recovered. In a second more serious case the urine was acid up to the 21st day, the pulse rose up to 120; then the urine commenced to become neutral, then alkaline; the nervous phenomena became more mild, the frequency of the pulse diminished; this state lasted from 10 to 11 days, during which time the urine flowed very copiously, became pale, and evinced a weak acid reaction. In two other cases the change of the acid into the alkaline reaction occurred before the 14th day of the disease, and in one of these cases the urine was so very intensely impregnated with carbonate of ammonia, and had so very stinking a smell, that it extended entirely over the sick chamber; the urine deposited a considerable pus-like or mucus-like sediment, consisting of phosphate of lime and phosphate of magnesia, and when mixed with acids effervesced violently; not till fourteen days after, and in the last case three weeks, did the urine evince an alkaline or neutral reaction, the acid reaction then gradually became established, and both parties recovered. It is deserving of notice that a secretion of urate of ammonia not unfrequently precedes the occurrence of the alkaline reaction and the appearance of the earthy phosphates in the urine, which, as Schönlein remarks, is in some measure to be considered as the precursor of the favorable change in the urine, and as the first critical effort of nature. The last time, when the process of the typhous disease appeared in a much milder form, this peculiar change of the reaction of the urine was in like manner observed several times, so that after the alkaline reaction has preceded and continued for some time, the recurrence of the acid reaction, combined with the clear appearance of the urine and a more copious secretion of the fluid, may be looked on as a favorable sign for the successful solution of the disease, whilst on the contrary, I remember some cases since the year 1840 where the urine came to have a neutral or alkaline reaction, but passed soon again into the acid reaction, in order to become alkaline again at a subsequent period, but in like manner only for a very short time: in one of these cases the disease was extremely tedious, and terminated fatally.—*Provincial Medical Journal, from Beiträge Zur Physiologischen und Pathologischen, Chemie Mikroskopie.*

### URINE IN SCARLATINA.

By Dr. Simon, of Berlin.

It is very incumbent on the rational physician to observe the urine in scarlet fever. It is well known how often the dropsy consecutive on scarlatina is indicated by the albuminous contents of the urine: however, I must observe that dropsy takes place after scarlatina without albuminuria, and that, under certain circumstances, though rarely, albuminuria occurs without dropsy following it. But in the case of scarlatina another phenomenon also merits attention—one which has been repeatedly pointed out by Schönlein, and by which the desquamation of the inner mucous membranes is indicated. The signs of desquamation of the mucous membrane of the bladder after scarlatina I have constantly observed, and it seems so much the more necessary that attention should be directed to this interesting phenomenon, and one so important with respect to the course of the disease, as in several cases the desquamation of the mucous membranes precedes that of the external skin; and, accordingly, the physician's attention is called to a period of this disease of so much importance. The urine becomes somewhat turbid, and after a length of time throws down a mucous sediment. When this is observed with the microscope, there are observed floating about portions of epithelium, partly single, and partly combined into several shreds. With these signs of desquamation of the bladder I have, moreover, observed those of severe irritation of this mucous membrane in the form of mucus-corpuscles. The cases where I saw albuminuria take place in adults after scarlatina are but few, and I cannot decide whether the albuminuria coincided with this process of desquamation. Another species of desquamation of the internal mucous membrane I have, a little while since, observed in the wards of Dr. Schönlein. A young girl, in whom desquamation of the outer skin, simultaneous with desquamation of the mucous membrane of the bladder, without, however, severe irritation of the kidneys, had occurred, complained of irritation in the organs of deglutition and in the trachea; she coughed up much mucus, and in the spitting-glass there appeared a purulent-looking sediment, which, on more minute examination with the microscope, showed itself as composed of numberless partly single portions, and partly of epithelial fragments united in shreds.

I obtained from Dr. Braun, of Berlin, a bloody urine for examination, with the question—whether its physical and chemical qualities were such as would warrant us in inferring the existence of scarlatina? The urine deposited a considerable sediment of a blood-red color, in which four different forms might be recognised—namely, numerous blood-corpuscles, a very large quantity of those cylindrical forms described in Bright's disease, mucus-corpuscles, and a large mass of uric acid, separated partly in rhombic crystals, partly in rather large round globules. The cylinders were of smaller width than I had observed them on a former occasion, and all of them were filled with a yellowish, dark, as it were, crystalline contents, which, in their outer appearance with respect to color and form bore some resemblance to uric acid separated in globules. These globules themselves might have been taken for the so-called inflammation-globules; they differ from them, however, in this, that on pressure they are commonly broken up into four or more globular fragments; some of the cylinders also admit of being crushed in this way, and from the character of the fragments I concluded with greater probability that the contents were a yellow-colored uric acid. The light red colored urine, which became clear over the sediment, contained a considerable quantity of albumen, and indeed much more than could be ascribed to the blood-serum. From this microscopical and chemical examination of the urine it was quite impossible to determine with certainty on the part of the chemist whether the irritation of the kidneys was the consequence of scarlatina; all that could be said with anything like conviction was that a very violent irritation of the kidneys was present. Dr. Braun was kind enough to make to me the following communication regarding the case itself. A boy, six years old, of a decidedly scrofulous habit of body, voided a blood-colored urine one night, after having first complained of headache during the day; the physician who was called in finds the patient in a low fever, and because his experience of late had led him to observe hæmaturia renalis in children merely as an ordinary precursor or accompanier of scarlatina, he examines him for this disease; however, neither eruptions nor angina, neither desquamation or œdema was observed; still the local examination confirmed the existence of renal irritation as perceptible from the urine; the right renal region was very sensible on pressure, and from thence there was violent pain along the abdomen to the umbilicus. Leeches were prescribed, and mild derivatives were ordered. On the next day there was some improvement; the hæmaturia renalis continued for three days; then again an improvement took place. On the night from the twelfth to the thirteenth day of the disease there was again a passage of bloody urine, some of which was sent me for examination: great restlessness; acute hydrocephalus closed the scene on the sixteenth day. Shortly before death the urine flowed copiously, and free from blood; on the second appearance of the hæmaturia, shortly before death, the pain which accompanied it during the first days did not return. This case is certainly interesting; nor can I feel myself warranted in concluding whether there existed a degeneration of the kidneys, or whether the irritation of the kidneys was occasioned by scarlatina or by the great quantity of uric acid that was deposited.—*Ibid.*

